# Kissing Bond Damage Detection Based on the Non-Reciprocity of Nonlinear Guided Waves in CFRP-Reinforced Steel Plates

**DOI:** 10.3390/ma19091859

**Published:** 2026-05-01

**Authors:** Ruiqi Guan, Haoqi Zhang, Jiarui Deng, Qingping Kang, Kai Wang

**Affiliations:** 1 College of Civil Engineering, Huaqiao University, Xiamen 361021, China; ruiqi.guan@hqu.edu.cn; 2 School of Aerospace Engineering, Xiamen University, Xiamen 361005, China

**Keywords:** reciprocity, nonlinear guided waves, CFRP-reinforced steel structures, kissing bond damage

## Abstract

Kissing bond damage, as an early stage of debonding in CFRP-reinforced structures, severely threatens the integrity and performance of structures. However, conventional ultrasonic guided wave methods are not sensitive to this sort of damage due to the micro scale of the damage and the complexity of the wave at the interface. To address this problem, the reciprocity of nonlinear guided waves is proposed to identify this damage and a novel reciprocity index based on the correlation coefficient of kissing bond damage-induced second harmonic waves is developed. A finite element model is established and kissing bond in CFRP-reinforced steel plate is simulated with a novel method, which is close to the actual condition of the kissing bond and can help reveal the interaction between guided waves and the interface. Experimental tests are also carried out to verify the proposed method. In addition, to prove the high efficiency of the proposed method, correlation coefficients of directly received signals are calculated to compare with the proposed reciprocity index. Both simulation and experiment results illustrate that the reciprocity index rises as the length of kissing bond increases, while correlation coefficients of directly received signals did not show a monotonic trend as the damage length changes, reflecting the validity and high sensitivity of the proposed method in identifying and quantitatively evaluating kissing bond damage in CFRP bonded steel structures.

## 1. Introduction

CFRP laminates, with lightweight and high-strength characteristics, have been extensively employed for the retrofitting of civil steel infrastructure, such as bridges and high-rise buildings. Bonded to the structure with an adhesive agent such as epoxy, CFRP improves the bearing capacity of steel structures, which further extends the service life of the whole structure [1]. CFRP-reinforced steel structures may be subjected to different types of failure modes, among which the major threat is the debonding between CFRP and the steel components [2]. Many factors can induce debonding damage in the bonded structure, for example, fatigue load [3], harsh environmental effects [4] and an incorrect installation process [5].

To ensure the reliability and integrity of CFRP-reinforced structures, it is urgent to detect debonding damage efficiently and to realise early warnings of the damage. Kissing bond damage, as the early stage of debonding in CFRP-reinforced structures, has been defined as intimate mechanical contact between the adhesive and adherend counterparts [6]. Distinct from debonding damage, which can be detected by a series of conventional non-destructive testing methods, especially the widely adopted ultrasonic guided waves [7,8,9,10], the kissing bond damage is difficult to be identified due to its negligible disturbance on the guided waves. Fortunately, ultrasonic guided waves with nonlinear characteristics have been found to be sensitive to micro-scale damage, including interfacial kissing bond. When a wave travels through the interface of the kissing bond, the contact interface opens and closes under different phases of the wave. This so-called “breathing” behaviour induces contact acoustic nonlinearity (CAN) [11] in the structure and the CAN can be detected via varied approaches such as higher harmonic generation [12,13], subharmonic generation [14,15] and mixed frequency responses [16,17], etc. Nevertheless, these methods usually rely on the benchmark signal for damage identification and evaluation.

It is well-known that the linear features of elastic waves follow the reciprocity principle, which means that in an undamaged structure, the received wave at location B excited at location A is the same as the received wave at location A excited at location B, regardless of the shape of the structure [18]. In comparison, if a nonlinear feature exists in a structure that is asymmetric at the centre of the sensing network, it will break the reciprocity of opposite sensing paths, because the CAN highly depends on the amplitude of the wave at the nonlinear damage [19]. When the same amplitudes of excited waves travel from different distances and pass through the nonlinear damage, they will generate CAN at different amplitudes. The non-reciprocity characteristic of nonlinear guided waves provides a baseline-free detection alternative for the CAN. Scalerandi et al. [20] investigated the influence of localised nonlinearities on the reciprocity principle in concrete beams. They found that the extent of non-reciprocity highly depends on the location of the damage. Huang et al. extended the reciprocity-based method to the composite plates [21,22,23]. They successfully located the damage based on a reciprocity index considering both direct waves and edge reflections. Apart from using the reciprocity through active sensing, passive detection analysing the reciprocity of reconstructed Green’s function from ambient noise has also been conducted to detect fatigue cracks in aluminium plate [24]. Due to the anisotropic properties of CFRP and multiple materials, the wave propagation and modes are more complex in CFRP-reinforced steel structures. And limited studies have been carried out to identify the kissing bond in CFRP-reinforced steel structures based on the reciprocity. Li et al. [12] detected the debonding in CFRP-reinforced steel plates with both linear and nonlinear guided waves, but the damage identification highly depends on the benchmark signal. Inspired by the above research, we develop a quantitative evaluation method for the kissing bond in CFRP-reinforced steel structures and extract second harmonic waves instead of directly received waves to establish a new reciprocity index based on the non-reciprocity of CAN, which would be more sensitive to the complex CFRP-reinforced steel structures.

In this study, the second harmonic generation approach is adopted to detect the kissing bond damage in CFRP-reinforced steel plates, and a new reciprocity index based on the correlation coefficients of second harmonic waves is provided to quantitatively assess the length of the kissing bond damage. Both FE simulation and experimental tests are conducted to evaluate the effect of the length of the kissing bond on the CAN and to verify the proposed reciprocity index. This paper is arranged as follows: in Section 2, the new reciprocity index is developed for the evaluation of kissing bond damage-induced CAN based on the extracted fundamental and second harmonic waves; Section 3 presents the FE modelling and its results for the quantitative evaluation of kissing bond damage; followed by Section 4 demonstrating the experimental setup and experimental test results of kissing bond detection for CFRP-reinforced steel specimens with different lengths of damages. Discussions about the proposed method are illustrated in Section 5. The conclusions of this paper are finally drawn in Section 6.

## 2. Reciprocity Index for Damage-Induced CAN

When a probing wave travels through the nonlinear interface, the breathing behaviours of the interface are triggered, which induces the CAN. Analytical studies have investigated the mechanism of CAN and treated the nonlinear interface (e.g., fatigue crack) as a second source which generates higher harmonics under breathing behaviour [25]. This second source at the interface can be approximated to a concentrated force, whose magnitude can be obtained using the following equation.

(1) $$T=\int_{interface\;area}{-\tilde{\sigma }}^{0}\cdot {\vec{x}}_{1}dA\cdot e^{i\omega_{0}t}f\left(t\right)$$

where ${\tilde{\sigma }}^{0}$ is the stress tensor of the guided wave, ${\vec{x}}_{1}$ denotes the direction vector of wave propagation, $\omega_{0}$ is the angular frequency of the excited wave and f(t) is an indicator function with values of 1 and 0 during the open and closed period of the interface, respectively.

In a 3D scenario, the in-plane displacements of generated second harmonics by the nonlinear interface can be expressed by

(2) $$u_{2\omega_{0},x_{1}}^{m}=\frac{k_{m}}{4i}\frac{2T_{2\omega_{0}}\int U^{m}\left(x_{3}\right)dx_{3}}{hI_{mm}}U^{m}\left(x_{3}\right)\left[H_{0}^{2}\left(k_{m}r\right)-H_{1}^{2}\left(k_{m}r\right)\right]$$

where $u_{2\omega_{0},x_{1}}^{m}$ represents the in-plane displacement of the mth-order mode in the x_1_ direction (along the length of the specimen), $U^{m}\left(x_{3}\right)$ denotes the in-plane displacement of the mth-order mode in the thickness direction of the specimen, i is the imaginary unit, k_m_ is the wavenumber of the propagating wave at double frequency, h is half of the specimen thickness, $T_{2\omega_{0}}$ is the in-plane component of the interface-induced second source at double frequency, $H_{0}^{2}\left(k_{m}r\right)$ and $H_{1}^{2}\left(k_{m}r\right)$ are Hankel functions of the second kind at the 0th order and 1st order, respectively, and $I_{mm}$ is the energy carried by the mth-order wave mode.

The damage-induced guided waves with nonlinear characteristics will exhibit breaking of reciprocity properties, meaning that the generated second harmonic waves from the opposite sensing path will show significant differences. The non-reciprocity of nonlinear guided waves is caused by the excitation dependence of CAN. When the nonlinear interface is not at the middle of the sensing path, the excited waves from opposite directions propagate different distances to the nonlinear interface, showing different stress levels ${\tilde{\sigma }}^{0}$ at the interface, which results in different T and $T_{2\omega_{0}}$ in Equation (1) and Equation (2), respectively. Since the displacements of generated second harmonics $u_{2\omega_{0},x_{1}}^{m}$ are directly proportional to the in-plane component of the interface-induced second source at double frequency $T_{2\omega_{0}}$, the breaking of reciprocity will be observed in the generated second harmonics from opposite directions. A larger area of the nonlinear interface results in higher force T at the interface and more dissimilarity in the generated second harmonics from opposite directions. Therefore, to quantitively evaluate the CAN, a reciprocity index is proposed based on the correlation coefficient of displacements of extracted second harmonic waves and fundamental waves.

(3) $${RI}_{can}=\left(1-\frac{\int_{t_{1}}^{t_{2}}U_{AB}^{f_{1}}\left(t\right)U_{BA}^{f_{1}}\left(t\right)dt}{\sqrt{\int_{t_{1}}^{t_{2}}U_{AB}^{f_{1}}{\left(t\right)}^{2}dt\int_{t_{1}}^{t_{2}}U_{BA}^{f_{1}}{\left(t\right)}^{2}dt}}\right)+\left(1-\frac{\int_{t_{1}}^{t_{2}}U_{AB}^{f_{2}}\left(t\right)U_{BA}^{f_{2}}\left(t\right)dt}{\sqrt{\int_{t_{1}}^{t_{2}}U_{AB}^{f_{2}}{\left(t\right)}^{2}dt\int_{t_{1}}^{t_{2}}U_{BA}^{f_{2}}{\left(t\right)}^{2}dt}}\right)$$

where $U_{AB}^{f_{1}}\left(t\right)$ and $U_{BA}^{f_{1}}\left(t\right)$ denote the displacements of received signals at the fundamental frequency of sensing path AB and BA, respectively; $U_{AB}^{f_{2}}\left(t\right)$ and $U_{BA}^{f_{2}}\left(t\right)$ are the displacements of received signals at the double frequency of sensing path AB and BA, respectively; t_1_ and t_2_ define the time window for the selected signals.

## 3. FE Simulation Analysis

### 3.1. Simulation Modelling

**Figure 1:**
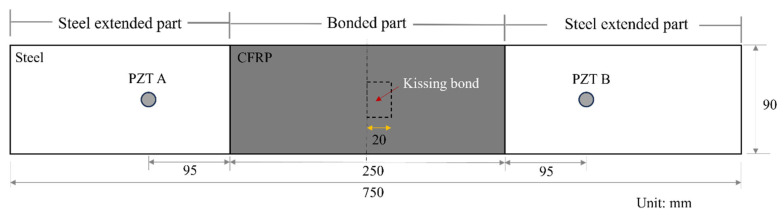
Schematic diagram of CFRP-reinforced steel plate model.

A 3D FE model in Abaqus/Explicit was established to simulate the kissing bond damage in CFRP-reinforced steel plate and its interaction with guided waves. The dimensions of the model and material properties of the steel and CFRP layer are shown in Figure 1 and Table 1, respectively.

The element type C3D8R is used for the model and the minimum element size in this model was set at 0.5 mm for both CFRP and steel layers, which is less than 1/20 of the wavelength of the received wave mode at the highest frequency of interest (450 kHz) [26]. Absorbing boundaries were set at four boundaries of the model, where the element type was changed to CIN3D8. To achieve accuracy in the calculation and convergence of the model, the time increment was fixed at 5 × 10^−8^ s.

**Figure 2:**
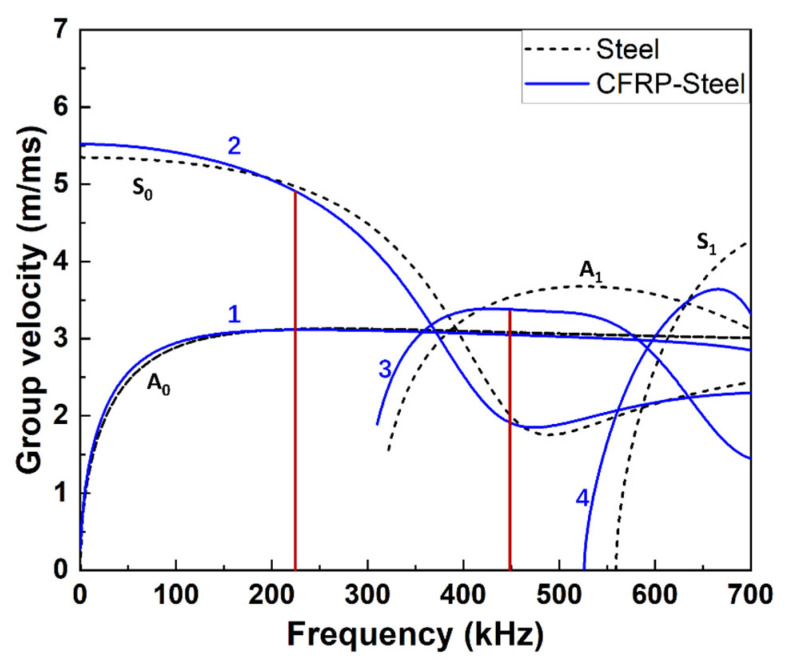
Dispersion curves of steel (5 mm) and CFRP (1 mm)–steel (5 mm). The red lines indicate the excitation wave Mode 2 at 225 kHz and the expected second harmonics at 450 kHz.

To select the excitation wave mode and frequency, dispersion curves of a CFRP–steel plate with the same thickness and material properties as the simulation model were plotted by the software Dispersion Calculator v3.1 [27] (as in Figure 2). With specific thicknesses (1 mm and 5 mm respectively in our study) and material properties of the CFRP and steel plate (as in Table 1), this software conducts theoretical calculations on the wave equation and obtains the dispersion curves of group velocity with different frequencies.

The dispersion curve of the 5 mm pure steel plate was also plotted in Figure 2 as a reference. Wave Mode 2 within the range of 200 to 300 kHz was suitable for wave excitation, since it has low dispersion and high group velocity. In this study, we only focused on CAN and to suppress the material nonlinearity in the CFRP-reinforced steel specimen, the matching of phase velocities of the excited wave mode and the second harmonic mode should not be satisfied [19]. In addition, the generated second harmonic wave should have low dispersion and the highest velocity at its frequency so that it can be easily extracted for further analysis. Considering the above conditions, an excitation at the frequency of 225 kHz was finally determined.

In the simulation model, an equivalent excitation was simulated, which is dynamic loads applied in the radial direction at nodes surrounding the expected location of circular PZT wafers. PZTs A and B were placed on the top of the steel plate at two ends of the specimen, 95 mm away from the CFRP layer. The excitation signals were 10-cycle Hanning-windowed tone bursts with a central frequency of 225 kHz. When PZT A acted as the actuator, PZT B would receive the signal, and vice versa. For the sensor receiving the signal, displacements in the direction of the length of the model were collected for further signal analysis.

For the bonding between the CFRP and steel layer, the bonded part was simulated using the “TIE” function in Abaqus 2016. Kissing bond damage was introduced between the CFRP and steel layer, deviating from the centre of the sensing path AB (as in Figure 1). At the location of the kissing bond, a surface-to-surface contact interaction was first set between the CFRP and steel layer and then a cohesive contact property with appropriate stiffness was assigned to the contact interface. The shear stiffness at the cohesive interface was set as a value small enough (less than 10% of the perfect bond condition using epoxy Araldite 420A and 420B in later experimental analysis). The parameters are set according to the combined experimental–computational modern methodologies based on Inverse Analysis [28,29], i.e., the kissing bond interface parameters are obtained from experiments and then assigned to the interface, so that the breathing effect of the interface can be simulated more accurately, resulting in CAN. When the tensile phase of the wave reaches the interface, the interface illustrates a low stiffness which is close to the condition of an open interface, and when the compressional phase of the wave passes through the interface, two surfaces of the interface will contact each other and the wave stress can travel through it. This finally induces the breathing behaviour of the interface, resulting in CAN. To quantitatively evaluate the kissing bond damage in the CFRP-reinforced steel plate, five models were established with different kissing bond lengths and one model as a benchmark was simulated with no kissing bond damage. The length of the kissing bond was changed from 10 mm to 30 mm with 5 mm increments and the width of the damage was kept at 20 mm for all the models.

**Figure 3:**
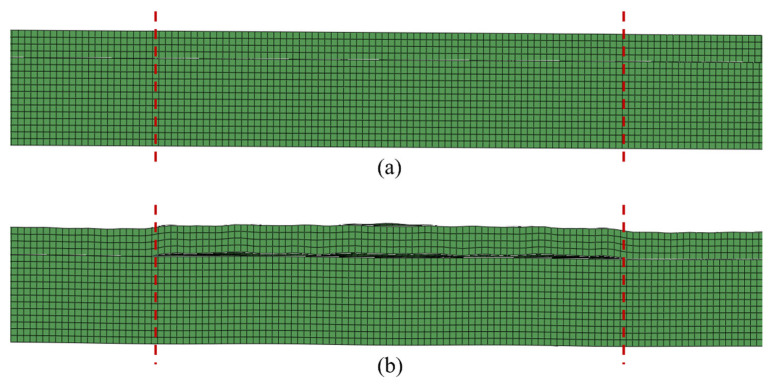
Deformation at the middle cross-section of the simulation model with 30 mm long kissing bond damage when the kissing bond interface between CFRP and steel is (**a**) closed at time step of 100 μs and (**b**) open at time step of 106 μs. The red dashed lines show the boundaries of the kissing bond at the cross-section.

The model field output was observed to confirm that the simulated kissing bond can introduce breathing behaviour, which is the dominant phenomenon for damage-induced CAN. Figure 3a,b show the typical zoomed cross-section deformations at different time steps in one of the simulation models which has 30 mm long kissing bond damage within the red dashed line boundaries.

The interface of the kissing bond exhibits an apparent open state in Figure 3b, when the wave could not travel through the interface. While when the contact interface is closed (Figure 3a), the wave could pass through the interface. As the wave travels through the interface, it is modulated by the dynamic breathing behaviour and generates higher harmonics. The deformation of the model indicates that this model successfully simulated the kissing bond, exhibiting a breathing effect at the interface which would generate CAN. Under different phases of the guided waves, this interface presents bilinear behaviour. It contacts and transmits stress under compression or high stiffness, while it separates and transmits no stress under tension or low stiffness. Compared with other methods for simulating nonlinear damage and debonding, such as using a seam crack or directly removing some elements at the interface, kissing bond damage simulated with this approach is close to its real condition and better reflects the interaction between guided waves and damage.

### 3.2. Simulation Results

**Figure 4:**
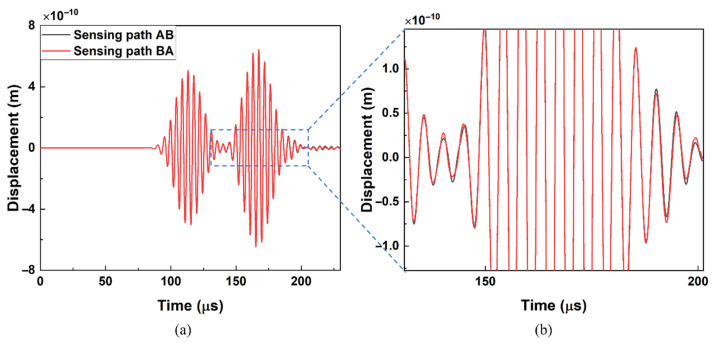
(**a**) Received time domain signals of the FE simulation model with 30 mm long kissing bond damage; (**b**) partial enlargement of (**a**).

The typical received signals from the simulation model with 30 mm long kissing bond damage are shown in Figure 4.

In Figure 4a, the first arrival wave packet is Mode 2 with the arrival time at 85 μs, which is close to the theoretical values (89 μs) calculated from the dispersion curve, and the second wave packet is Mode 1 with the arrival time at 140 μs, which is also similar to the theoretical values (142 μs). Signals excited at PZT A and PZT B show no significant difference in arrival time. In the zoomed-in Figure 4b, a slight difference in the shape of the waveform around the second wave packet can be observed. Nonlinearity in the model should break the reciprocity of received signals, but the kissing bond damage-induced CAN is too weak and has little distortion on the directly received time domain signals. Thus, further signal processing is needed to extract the nonlinear features for further quantitative evaluation.

**Figure 5:**
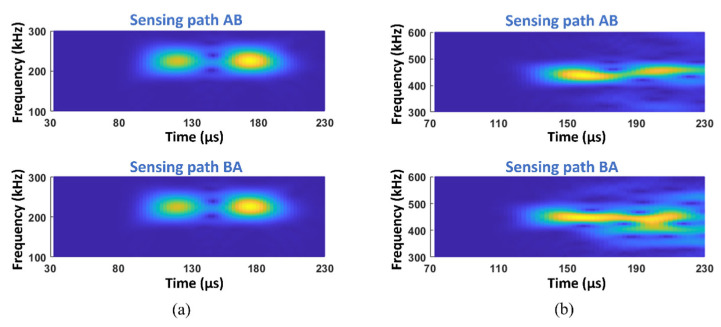
STFT spectra from sensing paths AB and BA of the simulation model with 30 mm length of kissing bond damage showing (**a**) the fundamental waves and (**b**) the second harmonic waves.

To extract the second harmonic waves and construct the reciprocity index to quantitatively evaluate the kissing bond damage, the original excited signal at PZT A was 180-degree phase-inversed and excited at the same location, and then the two received signals at PZT B were summed and processed by short-time Fourier transform (STFT), so that the fundamental waves can be eliminated and the second harmonic waves can be highlighted. The same procedure was applied to the sensing path BA. The directly received signal was also processed with STFT to compare the fundamental waves in the sensing paths AB and BA. The above signal processing method was executed on all the models with different kissing bond damages. Figure 5 shows STFT spectra of signals from sensing paths AB and BA in the model with 30 mm kissing bond damage.

For the fundamental waves, signals in opposite sensing paths still show no difference in time–frequency spectra, while the second harmonic waves show significant distinctions between sensing paths AB and BA.

**Figure 6:**
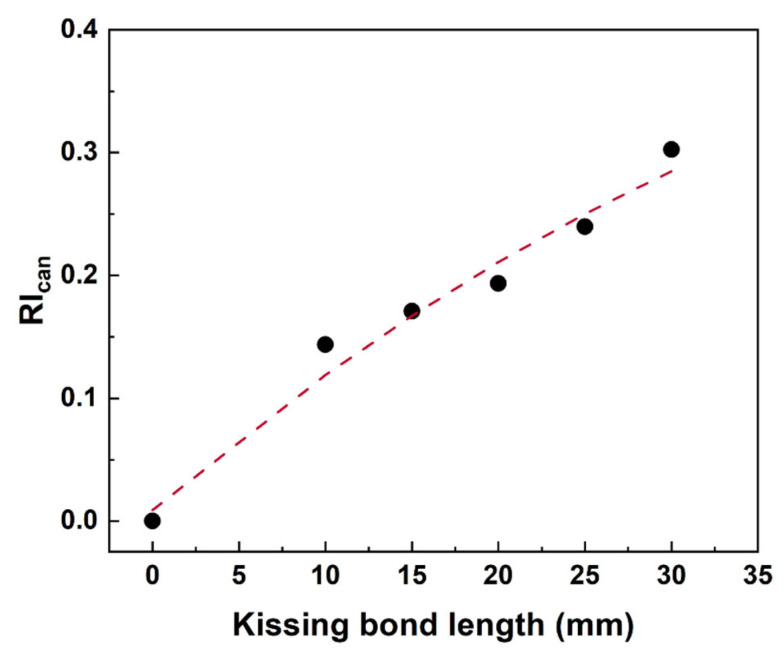
Plot of RI_can_ with kissing bond damage length from simulation results.

Signals at the fundamental frequency (225 kHz) and second harmonic frequency (450 kHz) were further extracted from the STFT spectra and the time domain signals of fundamental and second harmonic waves were obtained for reciprocity index calculation based on Equation (3). The results of RI_can_ for models with different lengths of kissing bond damages are listed in Table 2 and plotted in Figure 6 with different kissing bond lengths.

Correlation coefficients (CCs) of directly received signals, fundamental waves and second harmonic waves are also listed in the table to verify the efficiency of the proposed RI_can_.

In Table 2, the CCs of directly received signals change slightly with the kissing bond damage, but the variations are minimal and the severity of kissing bond damage cannot be assessed based on them. Thus, the previous proposed reciprocity index based on the corelation coefficients of directly received waves are not sensitive enough in this case. Dramatic changes in correlation coefficients of second harmonic waves with different lengths of kissing bond damages are observed, indicating that the extent of breaking the reciprocity is stronger with the increase in damage length. Therefore, the proposed RI_can_ based on extracted second harmonic waves can reflect the severity of damage more intuitively, as shown in Figure 6, increasing apparently with the length of kissing bond damage. It should be noted that the reciprocity of fundamental waves would also change with the CAN, but it is too marginal to be detected. It is included in the RI_can_ in case stronger CAN appears in experimental testing. In addition, since a damage evaluation method based on reciprocity is theoretically baseline-free, the results of the benchmark are only obtained to confirm the feasibility of this method, and the results (RI_can_ = 0) agree well with the theoretical expectation.

## 4. Experimental Test

### 4.1. Specimen Preparation and Experiment Setup

CFRP-reinforced steel plate specimens were prepared for experimental tests. One of the specimens, the benchmark, was a steel plate perfectly bonded with CFRP using epoxy, and for the other specimens, kissing bonds were introduced between CFRPs and the steel plates. The preparation procedures of these specimens were as follows: First, the surface of steel plates was sandblasted and cleaned with acetone. Second, the mould release agent (Loctite 770NC) was brushed on the steel plates within the expected kissing bond regions, and they were left to dry for five minutes. Then, epoxy (Araldite 2015A and 2015B) was brushed on the whole bonded zone on the steel plate, followed by a CFRP plate attached on top of the steel plate. Finally, heavy objects were placed on the specimen until the epoxy cured after 24 h. This method, using release agent to introduce damage in the specimen, has been reported can successfully simulate kissing bond damage, which is difficult to be identified by conventional ultrasonic testing method [30]. All the specimens in experimental testing have the same dimensions and material properties as the models in FE simulation analysis.

**Figure 7:**
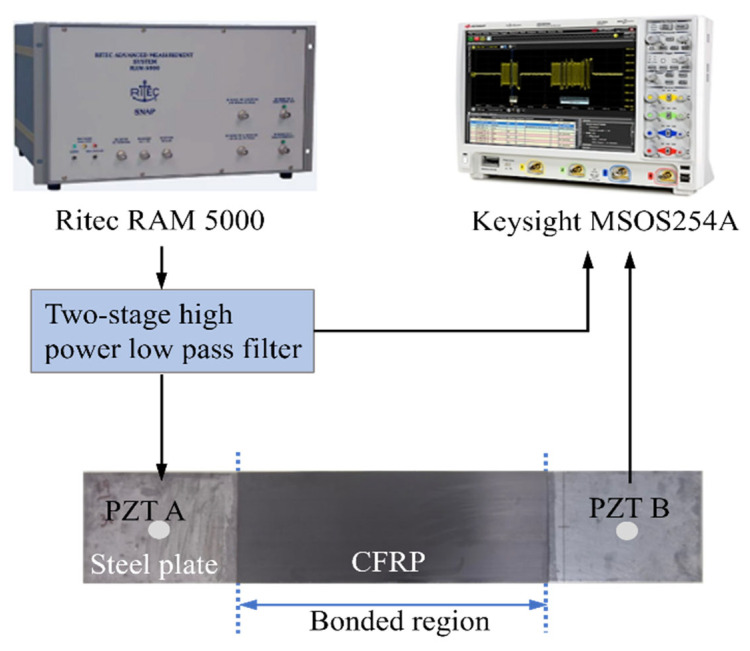
The signal generation and acquisition system in experimental test.

The system of signal generation and acquisition for experimental testing is illustrated in Figure 7.

A computer-controlled system (RAM 5000 SNAP, RITEC, Rochester, NY, USA) designed for nonlinear ultrasonic technique was used to generate signals which were 10-cycle Hanning-window modulated sinusoidal mixing tone bursts at excited voltage of 300V_p-p_, and these were sent to a PZT actuator (diameter 10 mm), exciting guided waves in the specimen. Two PZT wafers were attached to each of the specimens at the location shown in Figure 1. PZT A and PZT B acted as actuators in turn and signals from opposite directions were received. In addition, at each excitation, the same phase-inversion signal processing method as the simulation analysis was adopted for extracting the second harmonic waves in later signal processing. Wave signals acquired by PZT A or PZT B were averaged 1024 times to reduce the environment noises and were then collected by the oscilloscope (MSOS254A, Keysight, Santa Rosa, CA, USA) at sampling frequency of 20 MHz. All the received signals were further processed with the same method as the simulation.

### 4.2. Experiment Results

**Figure 8:**
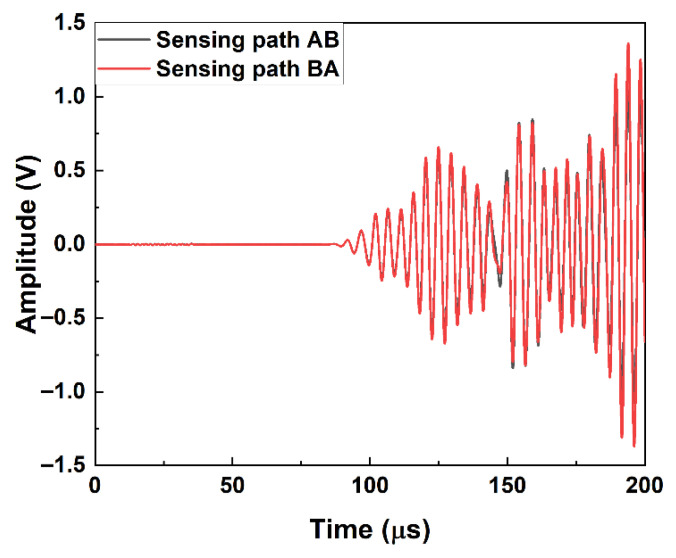
Received time domain signals of specimen with 30 mm long kissing bond damage.

One of the typical received signals from the specimen with 30 mm long kissing bond damage is shown in Figure 8.

Although the shapes of the wave packet are different to those in simulation results due to the boundary reflections in the specimen in the experiment, the arrival times of the first wave packet is at 84 μs, which is similar to the simulation results and should be the wave Mode 2. There is still no significant difference between the signals from sensing paths AB and BA. A small distinction between these two signals can only be observed between 140 μs and 150 μs.

**Figure 9:**
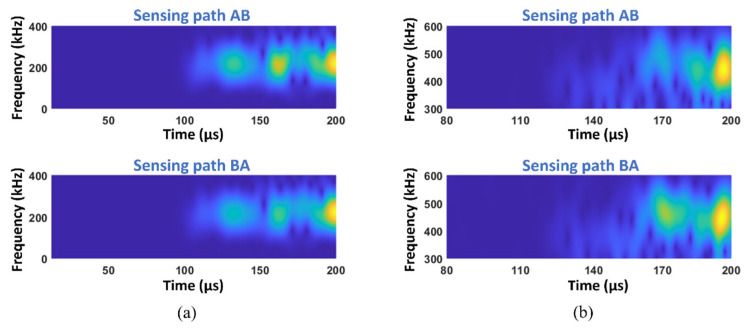
STFT spectra from sensing paths AB and BA of the specimen with 30 mm length of kissing bond damage showing (**a**) the fundamental waves and (**b**) the second harmonic waves.

After processing the received signals with the same procedure as the simulation, STFT spectra in Figure 9 were obtained.

Figure 9a shows the STFT spectra of fundamental waves from sensing paths AB and BA, respectively. These spectra are similar but the spectra amplitudes at 160 μs are different; a larger amplitude of the signals from sensing path AB can be observed. The slight difference in fundamental waves of opposite sensing paths is probably caused by the nonlinearity of the specimen. For the second harmonic wave STFT spectra in Figure 9b, apparent amplitude distributions can be observed in opposite sensing paths, implying the breaking of reciprocity of signals.

CCs of directly received signals, fundamental waves and second harmonic waves, along with RI_can_, are calculated and listed in Table 3 to compare the specimens with different lengths of kissing bond damages.

**Figure 10:**
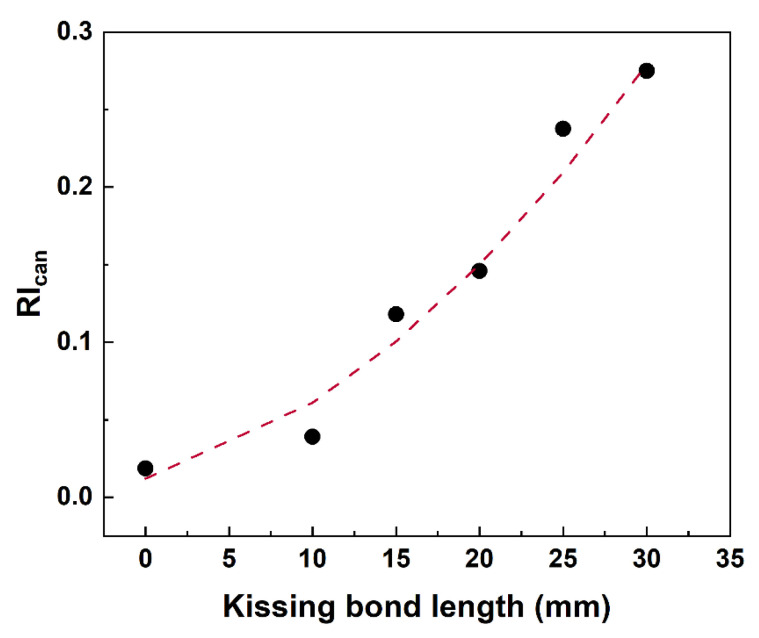
Plot of RI_can_ with kissing bond damage length.

Unlike the simulation results, variations in CCs of directly received signals are more obvious in specimens with different lengths of kissing bond damages. But using these values cannot distinguish the benchmark with 10 mm damage length specimen, as well as the 15 mm and 20 mm damage specimens. CCs of fundamental waves show an extent of non-reciprocity compared with the simulation results, but they do not monotonically decrease with the increase in kissing bond length. Thus, the variation in these values was caused by both CAN and material nonlinearity, and the latter one does not change with the damage length, reflecting the deviation of different specimens due to manual fabrication of the specimens. Although these values can be reduced using machine manufacturing for specimen preparation, it is still acceptable to include the CCs of fundamental waves because the material nonlinearity is marginal compared with CAN. This can be proved by the monotonically decreasing values of second harmonic waves and damage length. The proposed RI_can_, which comprehensively considers the reciprocity of fundamental and second harmonic waves, rises dramatically as the length of kissing bond damage increases (in Figure 10).

**Figure 11:**
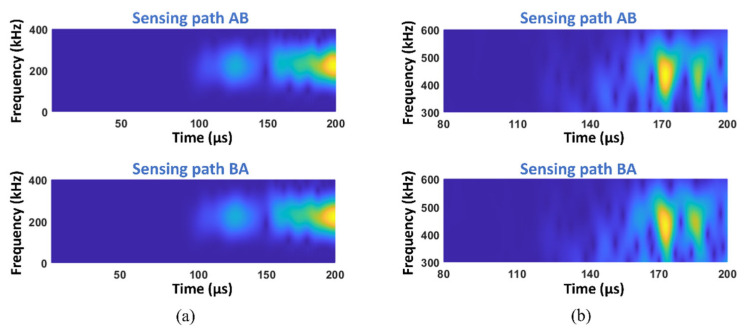
STFT spectra from sensing paths AB and BA of the intact specimen showing (**a**) the fundamental waves and (**b**) the second harmonic waves.

It is also noted that the RI_can_ of the specimen without damage is not zero and CCs of fundamental and second harmonic waves are 0.9952 and 0.9862, respectively. The STFT spectra of this benchmark are shown in Figure 11.

The difference between signals from opposite sensing paths in the second harmonic wave spectra can be observed around 190 μs. This again confirms that the material nonlinearity exists in the specimen and it is not evident in the early arrived signals but appears after a few reflections. The non-reciprocity caused by nonlinearity is weak in the benchmark, so the kissing bond damage can still be evaluated using the proposed RI_can_. In practice, standardised mechanical manufacturing of the specimens can realise the baseline-free evaluation based on the proposed RI_can_.

It is worth noting that damping behaviour may indeed affect the non-reciprocity of CAN. However, the proposed RI_can_ calculated the correlation coefficients of fundamental frequency and the second harmonic separately. Therefore, in experimental tests, the correlation coefficient of fundamental waves can be used to assess whether the damping in the opposite sensing paths is symmetric. Then, the correlation coefficient of second harmonic waves can be used to determine whether the observed non-reciprocity is induced by CAN. For all tested specimens, the correlation coefficients at the fundamental frequency ranged between 0.97 and 1.00, indicating that the influence of damping on system non-reciprocity is minimal. In contrast, the correlation coefficients of the second harmonic waves were apparently lower than those of the fundamental waves, reflecting that the non-reciprocity is predominantly caused by CAN. Experimental results indicate that damping behaviour has a negligible effect on the quantitative assessment of the kissing bond damage. Therefore, damping effects were not considered in the numerical simulations.

## 5. Discussion

Theoretically, kissing bond damage that is perfectly symmetric with respect to the centre of the sensing path cannot cause the breaking of signal reciprocity. Therefore, in this study the kissing bond damage was not designed exactly at the centre of the specimen. However, in practice, kissing bond damage is usually not perfectly symmetric, possessing non-uniformly distributed cohesive strength at the interface. It can still be evaluated using the proposed RI_can_, even if it is located at the centre of the sensing path. The existing model can better present the bilinear behaviour of the interface under guided wave modulation. The interface contacts and transmits wave stress under high stiffness, while it opens and blocks stress under low stiffness. The existing model can be extended to further simulate the non-uniform distribution of bond strength along the interface. For example, it can define key strength parameters of the contact surface, such as maximum traction and fracture energy, as functions of spatial coordinates, thereby simulating the inhomogeneity of interfacial bond strength.

When calculating RI_can_, the simulation and experimental test in this study adopted the same signal duration (200 μs), which theoretically corresponds to the end of Mode 1 (as shown in Figure 4). Only the inevitable reflections from the upper and lower boundaries of the specimen in the experiment were involved. Taking into account that the signal becomes complex after multiple reflections, making it difficult to investigate whether the breaking of reciprocity is entirely due to CAN, longer signals containing more reflections were not considered for RI_can_ calculation. The influence of including longer reflected signals on RI_can_ requires further investigation.

It should be noted that in practice, as the damage size increases to a certain extent and becomes linear damage, its RI_can_ will decrease. Therefore, the RI_can_ used in this study is only applicable to CAN induced by nonlinear damage. When it comes to linear damage, it may be more reasonable to calculate the RI_can_ by integrating the correlation coefficient of fundamental and second harmonic waves. Furthermore, the RI_can_ does not consider the weighting factor of the correlation coefficients of the fundamental wave and the second harmonic wave. This is mainly because damage induces non-reciprocity in both the fundamental and second harmonic waves in opposite sensing paths, but its effect on the non-reciprocity of the harmonic wave is more significant. Meanwhile, the non-reciprocities of linear systems such as damping change, boundary condition variation and coupling of the sensors, are also reflected in the fundamental wave. A more systematic investigation will be conducted in a future study to evaluate the influence of the asymmetry of linear systems and kissing bond damage on the weighting factors of correlation coefficients of fundamental and second harmonic waves.

## 6. Conclusions

The non-reciprocity of nonlinear guided waves was adopted to quantitatively evaluate the length of kissing bond damage in CFRP-reinforced steel plates. The contributions of this study can be summarised as follows:

- A novel reciprocity index, RI_can_, based on extracted second harmonic waves was established. It can be used to assess the kissing bond damage length from 10 mm to 30 mm.
- A new simulation method of kissing bond damage in the CFRP-reinforced steel structures was provided, which was close to the real condition of kissing bond damage and can better simulate the interaction between the guided wave and damage.
- High-sensitivity evaluation of CAN without a baseline signal was realised in the CFRP-reinforced steel structures using the proposed RI_can_.

In this study, both an FE simulation and experimental test were carried out to verify the efficiency of RI_can_. The simulation results showed that the proposed RI_can_ increases with the damage length, proving the validation of the proposed method. In addition, RI_can_ performs more sensitively to the damage length compared with previous methods which used a reciprocity index based on correlation coefficients of directly received signals. The RI_can_ decreases monotonically with the extend of kissing bond damage length from 10 mm to 30 mm; while using the previous correlation coefficients of directly received signals, it cannot tell the different between 10 mm and 15 mm damage, as well as the 20 mm and 25 mm damage. In the experimental test, the proposed method also successfully assessed the severity of the damage, regardless of deviation in the manual manufacturing of specimens; the RI_can_ also drops monotonically with the increase in damage length, but correlation coefficients of directly received signals show no significant trend with the changes in kissing bond damage. This study provided a baseline-free alternative with high sensitivity to evaluate the CAN induced by kissing bond damage instead of using higher harmonics directly. Using the proposed RI_can_, more systematic analysis considering the time information of received signals can be carried out to further realise the imaging of the kissing bond damage.

## Figures and Tables

**Table 1:** Material properties of CFRP and steel plate.

**Steel**	**Density (kg/m^3^)**				**Young’s Modulus (GPa)**			**Poisson’s Ratio**		
	7850				200			0.33		
**CFRP**	**Density** **(kg/m^3^)**	**E_1_** **(GPa)**	**E_2_** **(GPa)**	**E_3_** **(GPa)**	**Nu_12_**	**Nu_13_**	**Nu_23_**	**G_12_** **(GPa)**	**G_13_** **(GPa)**	**G_23_** **(GPa)**
	1550	120	8.8	8.8	0.33	0.33	0.45	8.2	8.2	3.0

**Table 2:** CCs of directly received signals, fundamental waves and second harmonic waves with different lengths of kissing bond damages.

	Benchmark	10 mm	15 mm	20 mm	25 mm	30 mm
CCs of directly received signals	1.0000	1.0000	1.0000	0.9999	0.999	0.9998
CCs of fundamental waves	1.0000	1.0000	1.0000	1.0000	1.0000	1.0000
CCs of second harmonic waves	1.0000	0.8564	0.8290	0.8064	0.7603	0.6976
RI_can_	0.0000	0.1436	0.1710	0.1936	0.2397	0.3024

**Table 3:** CCs of directly received signals, fundamental waves and second harmonic waves with different lengths of kissing bond damages in experiment specimens.

	Benchmark	10 mm	15 mm	20 mm	25 mm	30 mm
CCs of directly received signals	0.9954	0.9954	0.9842	0.9853	0.9838	0.9731
CCs of fundamental waves	0.9952	0.9978	0.9856	0.9785	0.9656	0.9698
CCs of second harmonic waves	0.9862	0.9631	0.8964	0.8755	0.7969	0.7553
RI_can_	0.0186	0.0391	0.118	0.146	0.2375	0.2749

## Data Availability

The original contributions presented in this study are included in the article. Further inquiries can be directed to the corresponding author.

## Nomenclature

CAN	contact acoustic nonlinearity
T	concentrated force at nonlinear interface
${\tilde{\sigma }}^{0}$	stress tensor of the guided wave
${\vec{x}}_{1}$	the direction vector of wave propagation
$\omega_{0}$	angular frequency of excited wave
$u_{2\omega_{0},x_{1}}^{m}$	in-plane displacement of the mth-order mode at double frequency along the length of the specimen
$U^{m}\left(x_{3}\right)$	in-plane displacement of the mth-order mode in the thickness direction of the specimen
i	the imaginary unit
k_m_	wavenumber of the propagating wave at double frequency
h	half of the specimen thickness
$T_{2\omega_{0}}$	in-plane component of the interface-induced second source at double frequency
$H_{0}^{2}\left(k_{m}r\right)$	Hankel function of the second kind at 0th order
$H_{1}^{2}\left(k_{m}r\right)$	Hankel function of the second kind at 1st order
$I_{mm}$	energy carried by the mth-order wave mode
${RI}_{can}$	reciprocity index
$U_{AB}^{f_{1}}\left(t\right)$	the displacements of received signals at fundamental frequency of sensing path AB
$U_{BA}^{f_{1}}\left(t\right)$	the displacements of received signals at fundamental frequency of sensing path BA
$U_{AB}^{f_{2}}\left(t\right)$	the displacements of received signals at double frequency of sensing path AB
$U_{BA}^{f_{2}}\left(t\right)$	the displacements of received signals at double frequency of sensing path BA
STFT	short-time Fourier transform
CC	correlation coefficient
